# Vitamin D and the promoter methylation of its metabolic pathway genes in association with the risk and prognosis of tuberculosis

**DOI:** 10.1186/s13148-018-0552-6

**Published:** 2018-09-12

**Authors:** Min Wang, Weimin Kong, Biyu He, Zhongqi Li, Huan Song, Peiyi Shi, Jianming Wang

**Affiliations:** 1 Department of Epidemiology, School of Public Health, Nanjing Medical University, 101 Longmian Ave, Nanjing, 211166 People’s Republic of China; 2 Department of Preventive Health Care, People’s Hospital of Suzhou High-tech Zone, Suzhou, People’s Republic of China; 3 Department of Nursing, The First People’s Hospital of Yancheng City, Yancheng, People’s Republic of China; 4 Key Laboratory of Infectious Diseases, School of Public Health, Nanjing Medical University, Nanjing, People’s Republic of China

**Keywords:** Tuberculosis, DNA methylation, Vitamin D, Risk, Prognosis

## Abstract

**Background:**

A variety of abnormalities in vitamin D metabolism have been reported in patients with active tuberculosis. However, intervention trials have produced inconsistent results. We hypothesized that genetic and epigenetic changes in the key genes of the vitamin D metabolic pathway may partly explain the differences between studies.

**Methods:**

We performed a case-control study followed by a prospective cohort study. We recruited 122 patients with pulmonary tuberculosis and 118 healthy controls. The serum 25-hydroxyvitamin D and 1,25-dihydroxyvitamin D levels were measured. The methylation of the promoter regions of key genes in the vitamin D metabolic pathway (CYP24A1, CYP27A1, CYP27B1, CYP2R1, and VDR) was detected using the Illumina MiSeq platform. The specific methylation profiles were examined as epigenetic biomarkers. The sensitivity, specificity, and receiver operating characteristic (ROC) curves were used to estimate the predictive value of the biomarkers.

**Results:**

The baseline serum 25-hydroxyvitamin D and 1,25-dihydroxyvitamin D concentrations in the cases were significantly lower than those in the controls (51.60 ± 27.25 nmol/L vs. 117.50 ± 75.50 nmol/L, *Z* = − 8.515, *P* < 0.001; 82.63 ± 51.43 pmol/L vs. 94.02 ± 49.26 pmol/L, *Z* = − 2.165, *P* = 0.03). We sequenced 310 CpG sites in five candidate genes. After Bonferroni correction, there were 55 differentially methylated CpG sites between cases and controls; 41.5% were in the CYP27B1 gene, 31.7% were in the CYP24A1 gene, 14.7% were in the VDR gene, and 12.3% were in the CYP27A1 gene. When we designated the CpG sites that remained significant after the Bonferroni correction as the biomarkers, the area under the curve (AUC) for the cumulative methylation was 0.810 (95% CI 0.754–0.866). There was an interaction between CYP27A1 methylation level and 1,25-dihydroxyvitamin D concentration associated with the risk of TB (OR_interaction_ = 4.11, 95% CI 1.26–13.36, *P* = 0.019). The serum 1,25-dihydroxyvitamin D concentration at the end of the intensive treatment stage was related to a patient’s prognosis (*P* = 0.008). There were 23 CpG sites that were individually related to the treatment outcomes, but the relationships were not significant after the Bonferroni correction.

**Conclusion:**

Both serum vitamin D concentrations and the methylation levels of key genes in the vitamin D metabolic pathway are related to the risk and prognosis of tuberculosis.

**Electronic supplementary material:**

The online version of this article (10.1186/s13148-018-0552-6) contains supplementary material, which is available to authorized users.

## Background

Despite the widespread use of the Bacillus Calmette-Guérin (BCG) vaccine, *Mycobacterium tuberculosis* (M.tb) infection and active tuberculosis (TB) remain major public health threats [1, 2]. TB is the ninth leading cause of death worldwide and is the leading cause of death from a single infectious agent, ranking above HIV/AIDS. In 2016, 10.4 million people fell ill with TB and there were an estimated 1.3 million TB deaths among HIV-negative people and an additional 374,000 deaths among HIV-positive people [3].

Factors related to the risk of TB include low socioeconomic status, poor nutrition, traditional/cultural traits, tobacco smoking, contacts with sputum smear-positive index patients, and genetic susceptibility [4–8]. Studies have correlated vitamin D deficiency with susceptibility to TB since 1651 when vitamin deficiency was found to be associated with TB for the first time [9]. Moreover, Stead et al. have shown racial differences in the incidence of TB associated with the levels of 25-hydroxyvitamin D (25(OH)D). Recently, studies have reported that hypovitaminosis D results in lower antimycobacterial immunity [10, 11] and is related to increased risk of TB [12, 13]. In vitro studies have revealed that 1,25-dihydroxyvitamin D (1,25(OH)_2_D) enhances innate immunity by increasing the expression of antimicrobial peptides, including cathelicidin, and inducing the autophagy of infected cells, thus restricting the intracellular growth of M.tb in macrophages [14]. Some studies have shown that vitamin D supplementation during the intensive phase of antimicrobial treatment can increase sputum negative conversion rates [15, 16]. Vitamin D supplementation has been believed to be beneficial in the treatment of patients with TB in observational studies; however, results from clinical trials have been inconclusive [17]. Confounding factors and reverse causation may partly explain the inconsistencies [18], but individual variations in vitamin D metabolism and related variations in immune responses should not be neglected.

Previous studies have mainly focused on genetic polymorphisms of genes in the vitamin D metabolic pathway. With the development of molecular biology, the role of epigenetic traits in gene expression has received greater attention [19]. DNA methylation, which involves the addition of a methyl group to the cytosine in a CpG dinucleotide, is a key epigenetic trait related to a number of biological processes including genomic imprinting, X-chromosome inactivation, aging, and carcinogenesis [20]. Studies have shown that hypermethylation in the promoter region of the cytochrome P450 gene can silence the gene and affect the vitamin D activity [21], but the role of this hypermethylation in the risk of TB has not been systematically studied. Thus, we performed a molecular epidemiological study in a Chinese population aiming to explore the effect of aberrant DNA methylation in the vitamin D metabolic pathway on serum 25(OH)D and 1,25(OH)_2_D levels and to determine its relation to the risk and prognosis of pulmonary TB.

## Methods

### Study design and study population

This study used a mixed case-control and prospective cohort design. We recruited 122 patients with pulmonary TB from Zhenjiang and Lianyungang in the province of Jiangsu in China during 2014 and 2016. TB cases were group-matched (by sex and age) with 118 controls from a pool of individuals who participated in community-based health examination programs. Among these controls, individuals with a history of TB, malignancy, diabetes, and HIV were excluded. This study was approved by the Ethics Committee of Nanjing Medical University. After informed consent was obtained from all participants, questionnaires were used to collect demographic data. Venous blood samples were collected for vitamin D measurement and molecular analyses.

### Serum 25(OH)D and 1,25(OH)_2_D measurement

We measured the serum vitamin D using a 25-hydroxyvitamin D kit and a 1,25-dihydroxyvitamin D EIA (Immunodiagnostic Systems Limited, UK). The intra- and interassay coefficient of variation (CV) were < 9% for 25(OH)D and < 20% for 1,25(OH)_2_D. The minimum detection limits were 12 nmol/L for 25(OH)D and 6 pmol/L for 1,25(OH)_2_D.

### Methylation analysis

We selected five key genes (CYP24A1, CYP27A1, CYP27B1, CYP2R1, and VDR) in the vitamin D metabolic pathway and sequenced the CpG islands in the promoter region of the candidate genes using the Illumina MiSeq platform. The DNA was subjected to sodium bisulfite treatment using an EZ DNA Methylation™-GOLD Kit (Zymo Research, Orange, CA, USA) according to the manufacturer’s protocols. Primers were designed to amplify the regions of interest from the bisulfite-converted DNA (Table 1).

**Table 1:** Primers designed for multiplex PCR

Gene	Fragment	Forward primer	Reverse primer
CYP24A1	CYP24A1 _1	TAGAGGAGGGYGGAGTGGTTT	CACACCCRATAAACTCCRAACTTC
	CYP24A1 _2	GGAGATAATTTTTAGGAAGTTATGYGAAGTT	CACTTCAATCCAAACTAAAAATATCTAACTC
CYP27A1	CYP27A1 _1	TTGGTTTYGTGGGGGTAGAG	CACCRCRTCCCTCTCCTACAA
	CYP27A1 _2	GGAGGGTYGAGTAAAGGTTAGTTAGAT	AAAACCTATCCCRATATAAAACTTCC
	CYP27A1 _3	ATTTTGGGYGGGGGTGTAG	CCCTCCAAAAATCAAATAACTAACC
	CYP27A1 _4	TTTTYGGATTGATTTYGGAGTTAGT	ACTATACRTTTTCCRTACTATATTACTCTTTCC
	CYP27A1 _5	GGTTGAGATTAGATTTYGTAGATGATG	ACCAACTATACCATCCTACTAAATCCT
CYP27B1	CYP27B1 _1	GGTTGAGATATGATGTTTAGGAGAAG	TCCCTTCCTACCTACAACTCRTATA
	CYP27B1 _2	TTTGGYGTGGGTATAGGTTAAGTTG	CTCACRCAATAAACAATCCRCAAAC
	CYP27B1 _3	GAGTTGTTGYGATAGGAGGGATT	CAACCRACCTCCCACCA
CYP2R1	CYP2R1 _1	TTAATGGGAGTATGGTAGGGTTG	AAAAACCCATCRACCRCCTCTA
	CYP2R1 _2	GGTAGGGAGGGTYGTTAGGTTG	CAAAACTAAATCRCCTCRAAACCTC
	CYP2R1 _3	TGTAGGGGGAGTTTYGTTTTTGT	CAAACACCRAAAAACCTACTATTAACC
	CYP2R1 _4	GGAAAATTAAGGYGTTTTGAGTTTTA	CACACAAAAAACRCCTTTTAAATATCTAC
VDR	VDR _1	GTAGTTATTTATAATTTTAGGTTTTAGGAGGTAG	CTCAACCTAATCCCACAAATTAAAA
	VDR _2	AGGTGATATYGGGTGGGAGTAAT	CCACCTAAACTAACCAAACCAA
	VDR _3	GGTGTTAGTYGGTAGGYGTTTTTTAG	CATAAAACAAAACACRCTTCTACCCT
	VDR _4	TTTYGATTAATATAGGTTGAAGYGGGTA	CCCACAAATCCAATCCTCTC
	VDR _6	GAATTYGGGAGTAGYGGGAAAG	TACTAAACACTATATTAACRAAACATTTCTCC

Multiplex PCR was performed using the optimized primer sets. A 20-μl PCR reaction mixture was prepared for each reaction that included 2 μl of template DNA, 3 mM Mg^2+^, 0.2 mM dNTP, 0.1 μM of each primer, 1× buffer (Takara, Tokyo, Japan), and 1 U of HotStarTaq polymerase (Takara, Tokyo, Japan). The cycling program was 95 °C for 2 min; 11 cycles of 94 °C for 20 s, 63 °C for 40 s with a decreasing temperature step of 0.5 °C per cycle, and 72 °C for 1 min; 24 cycles of 94 °C for 20 s, 65 °C for 30 s, and 72 °C for 1 min; and 72 °C for 2 min.

PCR amplicons from different panels were quantified and pooled, diluted, and subjected to a second round of amplification using the indexed primers. A 20-μl mixture was prepared for each reaction that included 0.3 μM index primer, 0.3 μM forward primer, 0.3 mM dNTP, 1× buffer (New England Biolabs, MA, the USA), 1 U of Q5™ DNA polymerase (New England Biolabs), and 1 μL of diluted template. The cycling program was 98 °C for 30 s; 11 cycles of 98 °C for 10 s, 65 °C for 30 s, and 72 °C for 30 s; and 72 °C for 5 min. The PCR products were separated by agarose gel electrophoresis and purified using a QIAquick Gel Extraction Kit (Qiagen, Hilden, Germany). The libraries from the different samples were quantified and pooled together, then sequenced on the Illumina MiSeq platform according to the manufacturer’s protocols. Sequencing was performed with 2 × 300 bp (overall sequencing read length) paired-end runs.

### Statistical analysis

Data were entered with EpiData 3.1 software (Denmark) and analyzed using STATA 12.0 (StataCorp, College Station, TX, USA). Student’s *t* test and the chi-square test were used to compare the distributions of demographic variables and risk factors between cases and controls. Univariate and multivariate logistic regression models were used to calculate odds ratios (ORs) and 95% confidence intervals (CIs). We analyzed both individual and cumulative methylation levels of the candidate genes. The sensitivity, specificity, and the area under the receiver operating characteristic (ROC) curve were estimated to assess the diagnostic value of the biomarkers. A Spearman correlation was used to estimate the relationship between serum vitamin D levels and methylation status. A Kaplan-Meier curve was used to analyze the effect of methylation level on initial sputum conversion.

## Results

### General characteristics of study subjects

In total, 122 TB cases (78.7% males and 21.3% females) and 118 controls (72.9% males and 27.1% females) were involved in the analysis. The average age (± standard deviation, SD) was 50.83 ( 20.04) years in cases and 51.97 (± 12.49) years in controls. Due to the frequency matching, there was no significant difference in the distribution of age and sex between the two groups. However, the distribution of marital status was significantly different (*P* < 0.001). The proportion of those who had ever smoked in the cases was slightly higher than that in the controls (42.6% vs. 39.0%), while the percentage of those who drank alcohol was slightly lower in the cases than in the controls (26.2% vs. 29.7%), although there was not a significant difference (Table 2). Among the cases, 46 (37.7%) were cured, 61 (50.0%) completed treatment, and 15 (12.3%) failed to be treated. We categorized the cases into two groups: successful (cured or completed treatment) and unsuccessful.

**Table 2:** General characteristics of cases and controls

Variables	Controls (*n* = 118)	Cases (*n* = 122)	*t*/*χ*^2^	*P*
Age (years)				
Mean ± SD	51.97 ± 12.49	50.83 ± 20.04	0.530	0.597
Sex [*n*(%)]			1.104	0.293
Male	86 (72.9)	96 (78.7)		
Female	32 (27.1)	26 (21.3)		
Marital status [*n*(%)]				
Unmarried	3 (2.5)	25 (20.5)	18.777	< 0.001
Married	111 (94.1)	94 (77.0)		
Widowed/divorced	4 (3.4)	3 (2.5)		
Smoking [*n*(%)]			0.329	0.566
Never	72 (61.0)	70 (57.4)		
Ever	46 (39.0)	52 (42.6)		
Drinking [*n*(%)]			0.351	0.554
Never	83 (70.3)	90 (73.8)		
Ever	35 (29.7)	32 (26.2)		

### Serum vitamin D concentrations and methylation levels of CpG sites and the risk of TB

The baseline concentrations of serum 25(OH)D and 1,25(OH)_2_D were 51.60 ± 27.25 nmol/L and 82.63 ± 51.43 pmol/L among cases, respectively, which were significantly lower than those in the controls (25(OH)D 117.50 ± 75.50 nmol/L, *Z* = − 8.515, *P* < 0.001; 1,25(OH)_2_D 94.02 ± 49.26 pmol/L, *Z* = − 2.165, *P* = 0.03). The serum level of 1,25(OH)_2_D declined during the early phase of antituberculosis treatment, while at the end of the intensive treatment stage, it was 70.81 ± 44.50 pmol/L (*Z* = − 2.606, *P* = 0.009).

We sequenced 310 CpG sites in the promoter regions of the candidate genes (Table 3). The methylation levels of specific sites between cases and controls are shown in Table 4. The correlations for methylation levels in each region with 1,25(OH)_2_D concentrations were listed in Additional file 1. After Bonferroni correction, there were 55 differentially methylated CpG sites between cases and controls, 41.5% of which were in the CYP27B1 gene, 31.7% of which were in the CYP24A1 gene, 14.7% of which were in the VDR gene, and 12.3% of which were in the CYP27A1 gene. We calculated the cumulative methylation levels by adding the frequencies for all the CpG sites in each gene region and found that the methylation level of CYP27A1_3 was significantly associated with the level of serum 1,25(OH)_2_D (*P* = 0.045). To further analyze the cumulative methylation levels by considering multiple CpG sites in each gene, we constructed four models. In model 1, we calculated the cumulative methylation levels by adding the frequencies for all the CpG sites in each gene. In model 2, we calculated the cumulative methylation levels by adding the frequencies for the statistically significant CpG sites in each gene. Model 3 only included hypermethylated CpG sites in the cases and excluded CpG sites with inverse relations between cases and controls. Model 4 only included CpG sites that were statistically significant after the Bonferroni correction.

**Table 3:** Sequenced sites of selected genes

Gene	Fragment	Start/stop	Size(bp)	Number of CpG sites
CYP24A1	CYP24A1_1	52790591/52790815	224	13
	CYP24A1_2	52790767/52791019	252	28
CYP27A1	CYP27A1_1	219646982/219646721	261	22
	CYP27A1_2	219646810/219646561	249	22
	CYP27A1_3	219646624/219646403	221	18
	CYP27A1_4	219646465/219646204	261	8
	CYP27A1_5	219646286/219646037	249	3
CYP27B1	CYP27B1_1	58160882/58160619	263	16
	CYP27B1_2	58160053/58159785	268	23
	CYP27B1_3	58159890/58159688	202	14
CYP2R1	CYP2R1_1	14913830/14913634	196	14
	CYP2R1_2	14913505/14913273	232	15
	CYP2R1_3	14913339/14913061	278	24
	CYP2R1_4	14913116/14912845	271	15
VDR	VDR _1	48299590/48299323	267	18
	VDR _2	48299412/48299179	233	19
	VDR _3	48299247/48299017	230	13
	VDR _4	48299106/48298885	221	10
	VDR _6	48298733/48298464	269	15

**Table 4:** Methylation levels of specific sites between cases and controls

Gene	Fragment	Methylation level of controls (*n* = 118)		Methylation level of cases (*n* = 122)	
		Mean ± SD	Median	Mean ± SD	Median
CYP24A1	CYP24A1_1	0.39 ± 0.13	0.374	0.35 ± 0.12	0.335
	CYP24A1_2	0.48 ± 0.08	0.461	0.41 ± 0.07	0.397
CYP27A1	CYP27A1_1	0.87 ± 0.23	0.818	0.75 ± 0.27	0.678
	CYP27A1_2	0.78 ± 0.24	0.716	0.63 ± 0.24	0.557
	CYP27A1_3	0.54 ± 0.20	0.508	0.43 ± 0.18	0.398
	CYP27A1_4	0.72 ± 0.17	0.697	0.63 ± 0.12	0.606
	CYP27A1_5	1.79 ± 0.20	1.804	1.76 ± 0.01	1.784
CYP27B1	CYP27B1_1	3.22 ± 0.56	3.167	2.78 ± 0.56	2.664
	CYP27B1_2	1.56 ± 0.33	1.493	1.40 ± 0.32	1.345
	CYP27B1_3	0.60 ± 0.14	0.578	0.53 ± 0.14	0.499
CYP2R1	CYP2R1_1	0.13 ± 0.01	0.129	0.13 ± 0.01	0.126
	CYP2R1_2	0.15 ± 0.01	0.152	0.15 ± 0.01	0.148
	CYP2R1_3	0.22 ± 0.05	0.223	0.22 ± 0.05	0.213
	CYP2R1_4	0.13 ± 0.02	0.124	0.12 ± 0.01	0.123
VDR	VDR _1	0.94 ± 0.23	0.897	0.82 ± 0.23	0.781
	VDR _2	0.37 ± 0.05	0.362	0.33 ± 0.06	0.313
	VDR _3	0.21 ± 0.10	0.196	0.20 ± 0.10	0.188
	VDR _4	0.13 ± 0.02	0.132	0.12 ± 0.02	0.122
	VDR _6	0.26 ± 0.07	0.270	0.27 ± 0.07	0.263

#### Model 1

We calculated the cumulative methylation levels by adding the frequencies for all the CpG sites in each gene. The cumulative methylation levels of CYP24A1, CYP27A1, CYP27B1, CYP2R1, and VDR were significantly different between cases and controls (*P* < 0.05, Table 5). The area under the curve (AUC) for each gene is listed in Table 6. The cumulative methylation levels of the CYP27A1 gene showed the highest diagnostic value, with an AUC of 0.739 (95% CI 0.675–0.802), followed by CYP27B1 (AUC 0.735, 95% CI 0.671–0.799). The AUC obtained by combining the cumulative methylation levels of the CpG sites in all genotyped genes was 0.747 (95% CI 0.685–0.809).

**Table 5:** Cumulative methylation levels of multiple CpG sites in each gene between cases and controls using different models

Gene	Model 1				Model 2				Model 3				Model 4			
	Cumulative methylation level		*t*	*P*	Cumulative methylation level		*t*	*P*	Cumulative methylation level		*t*	*P*	Cumulative methylation level		*t*	*P*
	Controls (*n* = 118)	Cases (*n* = 122)			Controls (*n* = 118)	Cases (*n* = 122)			Controls (*n* = 118)	Cases (*n* = 122)			Controls (*n* = 118)	Cases (*n* = 122)		
CYP24A1	0.87 ± 0.16	0.76 ± 0.17	5.120	< 0.001	0.46 ± 0.08	0.38 ± 0.08	7.929	< 0.001	0.46 ± 0.08	0.38 ± 0.08	7.929	< 0.001	0.28 ± 0.06	0.23 ± 0.05	7.699	< 0.001
CYP27A1	4.70 ± 0.74	4.19 ± 0.68	5.537	< 0.001	2.40 ± 0.57	1.96 ± 0.51	6.182	< 0.001	2.40 ± 0.57	1.96 ± 0.51	6.182	< 0.001	0.59 ± 0.17	0.47 ± 0.16	5.803	< 0.001
CYP27B1	5.37 ± 0.97	4.72 ± 0.95	5.271	< 0.001	4.95 ± 0.92	4.31 ± 0.90	5.417	< 0.001	4.95 ± 0.92	4.31 ± 0.90	5.417	< 0.001	3.05 ± 0.61	2.57 ± 0.61	5.998	< 0.001
CYP2R1	0.63 ± 0.06	0.61 ± 0.06	2.118	0.035	0.08 ± 0.02	0.07 ± 0.02	2.203	0.029	0.07 ± 0.01	0.06 ± 0.01	6.207	< 0.001	–	–	–	–
VDR	1.92 ± 0.32	1.74 ± 0.32	4.203	< 0.001	1.12 ± 0.22	0.97 ± 0.23	4.909	< 0.001	1.04 ± 0.21	0.88 ± 0.22	5.812	< 0.001	0.40 ± 0.07	0.33 ± 0.08	6.237	< 0.001

**Table 6:** Diagnostic values for TB of selected genes using different models

Gene	Model 1			Model 2			Model 3			Model 4		
	AUC	95% CI	*P*	AUC	95% CI	*P*	AUC	95% CI	*P*	AUC	95% CI	*P*
CYP24A1	0.707	0.641–0.772	< 0.001	0.794	0.737–0.851	< 0.001	0.794	0.737–0.851	< 0.001	0.793	0.736–0.850	< 0.001
CYP27A1	0.739	0.675–0.802	< 0.001	0.760	0.699–0.821	< 0.001	0.760	0.699–0.821	< 0.001	0.747	0.685–0.809	< 0.001
CYP27B1	0.735	0.671–0.799	< 0.001	0.739	0.675–0.803	< 0.001	0.739	0.675–0.803	< 0.001	0.747	0.684–0.810	< 0.001
CYP2R1	0.578	0.506–0.650	0.037	0.611	0.540–0.683	0.003	0.743	0.679–0.806	< 0.001	–	–	–
VDR	0.664	0.596–0.732	< 0.001	0.709	0.644–0.775	< 0.001	0.740	0.677–0.803	< 0.001	0.758	0.696–0.819	< 0.001

*AUC* area under the curve, *CI* confidence interval

#### Model 2

We calculated the cumulative methylation levels by adding the frequencies for the statistically significant CpG sites in each gene. The cumulative methylation levels of CYP24A1, CYP27A1, CYP27B1, CYP2R1, and VDR remained significantly different between cases and controls (*P* < 0.05). As shown in Table 6, the AUC of each gene was higher than that in model 1. CYP24A1 showed the highest diagnostic value, with an AUC of 0.794 (95% CI 0.737–0.851). The AUC obtained by combining the cumulative methylation levels of the CpG sites in model 2 was 0.805 (95% CI 0.749–0.860).

#### Model 3

We further excluded CpG sites with inverse relations between cases and controls and only included 164 hypermethylated CpG sites in the cases for analysis. The cumulative methylation levels of CYP24A1, CYP27A1, CYP27B1, CYP2R1, and VDR remained significantly different between cases and controls (*P* < 0.001). The CpG sites of the CYP24A1, CYP27A1, and CYP27B1 genes included in model 3 were the same as those in model 2. For the CYP2R1 and VDR genes, the AUC increased to 0.743 (95% CI 0.679–0.806) and 0.740 (95% CI: 0.677–0.803), respectively (Table 6). The AUC obtained by combining the cumulative methylation levels of all of the aforementioned CpG sites was 0.838 (95% CI 0.789–0.888).

#### Model 4

We included 55 methylated CpG sites that remained statistically significant after the Bonferroni correction. As shown in Table 5, CYP24A1, CYP27A1, CYP27B1, and VDR had significantly different methylation levels between cases and controls (*P* < 0.001). The AUC obtained by combining the cumulative methylation levels of all the aforementioned CpG sites was 0.810 (95% CI 0.754–0.866).

### Interaction between serum vitamin D and methylation levels

As shown in Table 7, the methylation levels of the CYP24A1, CYP27A1, CYP27B1, and VDR genes were significantly associated with the levels of serum 25(OH)D (*P* < 0.05). To explore the interaction between vitamin D and methylation in the risk of TB, we categorized the genes into hyper- and hypomethylated genes based on the ROC curves and divided the serum vitamin D levels into low and high levels based on the median. There was a significant interaction between CYP27A1 methylation levels and 1,25(OH)_2_D concentrations in model 1 and model 4 and between the methylation levels of VDR and CYP2R1 and 1,25(OH)_2_D concentrations in model 3 (*P* < 0.05, Table 8). Based on model 4, the OR_interaction_ was 4.11 (95% CI 1.26–13.36, *P* = 0.019) (Table 9).

**Table 7:** Correlation analysis between cumulative methylation levels and 25-hydroxyvitamin D levels

Gene	Model 1		Model 2		Model 3		Model 4	
	r	*P*	r	*P*	r	*P*	r	*P*
CYP24A1	0.213	< 0.001	0.287	< 0.001	0.287	< 0.001	0.303	< 0.001
CYP27A1	0.294	< 0.001	0.279	< 0.001	0.279	< 0.001	0.227	< 0.001
CYP27B1	0.195	0.002	0.201	0.002	0.201	0.002	0.210	0.001
CYP2R1	0.021	0.742	0.078	0.232	0.231	< 0.001	–	–
VDR	0.177	0.006	0.202	0.002	0.238	< 0.001	0.308	< 0.001

*r* correlation coefficient

**Table 8:** Interaction analysis of CpG island methylation levels and 1,25-dihydroxyvitamin D levels in TB risk

Gene	Model 1			Model 2			Model 3			Model 4		
	OR	95% CI	*P*	OR	95% CI	*P*	OR	95% CI	*P*	OR	95% CI	*P*
VDR	1.99	0.68–5.84	0.209	2.72	0.89–8.30	0.078	3.51	1.11–11.12	0.033	2.45	0.78–7.68	0.125
CYP27B1	2.39	0.69–8.34	0.171	2.60	0.68–10.01	0.164	2.60	0.68–10.01	0.164	2.19	0.67–7.18	0.197
CYP24A1	1.79	0.51–6.29	0.367	2.52	0.75–8.47	0.136	2.52	0.75–8.47	0.136	2.39	0.69–8.29	0.169
CYP2R1	1.19	0.40–3.47	0.756	2.06	0.71–5.98	0.184	4.34	1.30–14.49	0.017	–	–	–
CYP27A1	3.49	1.10–11.08	0.034	2.47	0.77–7.98	0.129	2.47	0.77–7.98	0.129	4.11	1.26–13.36	0.019

**Table 9:** Crossover analysis of CYP27A1 methylation levels and 1,25-dihydroxyvitamin D levels in the risk of TB

1,25(OH)_2_D^a^	Cumulative methylation level^b^	Cases	Controls	OR	95% CI
Low	High	23	50	0.30	0.14–0.66
High	High	21	42	0.33	0.15–0.73
Low	Low	52	9	3.78	1.48–9.62
High	Low	26	17	1	
OR_interaction_ = 4.11 (95% CI 1.26–13.36, *P* = 0.019)					

^a^Cutoff point of 1,25(OH)_2_D 86 pmol/L

^b^Cutoff point of cumulative methylation level of CYP27A1 0.48

### Treatment outcomes of patients with TB

**Fig. 1:**
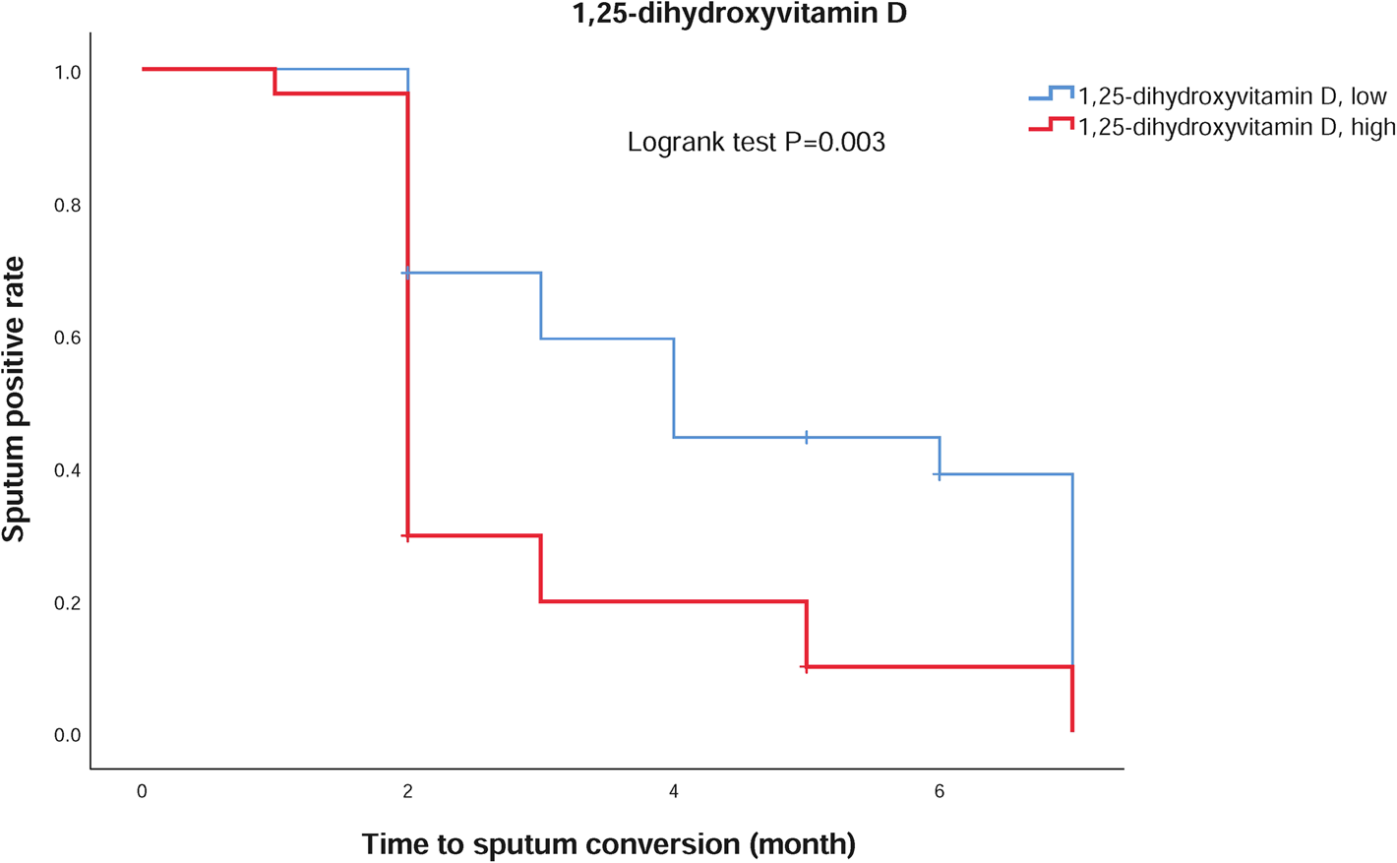
Time to sputum conversion in TB patients and 1,25-dihydroxyvitamin D concentration

The baseline serum 1,25(OH)_2_D concentration was related to the treatment outcome (*Z* = − 2.655, *P* = 0.008). Patients with high serum 1,25(OH)_2_D concentrations had a decreased risk of treatment failure (adjusted relative risk (RR) 0.07, 95% CI 0.01–0.39). As shown in Fig. 1, the sputum conversion rate was significantly higher in patients with higher serum 1,25(OH)_2_D levels (χ^2^ = 8.85, *P* = 0.003).

**Fig. 2:**
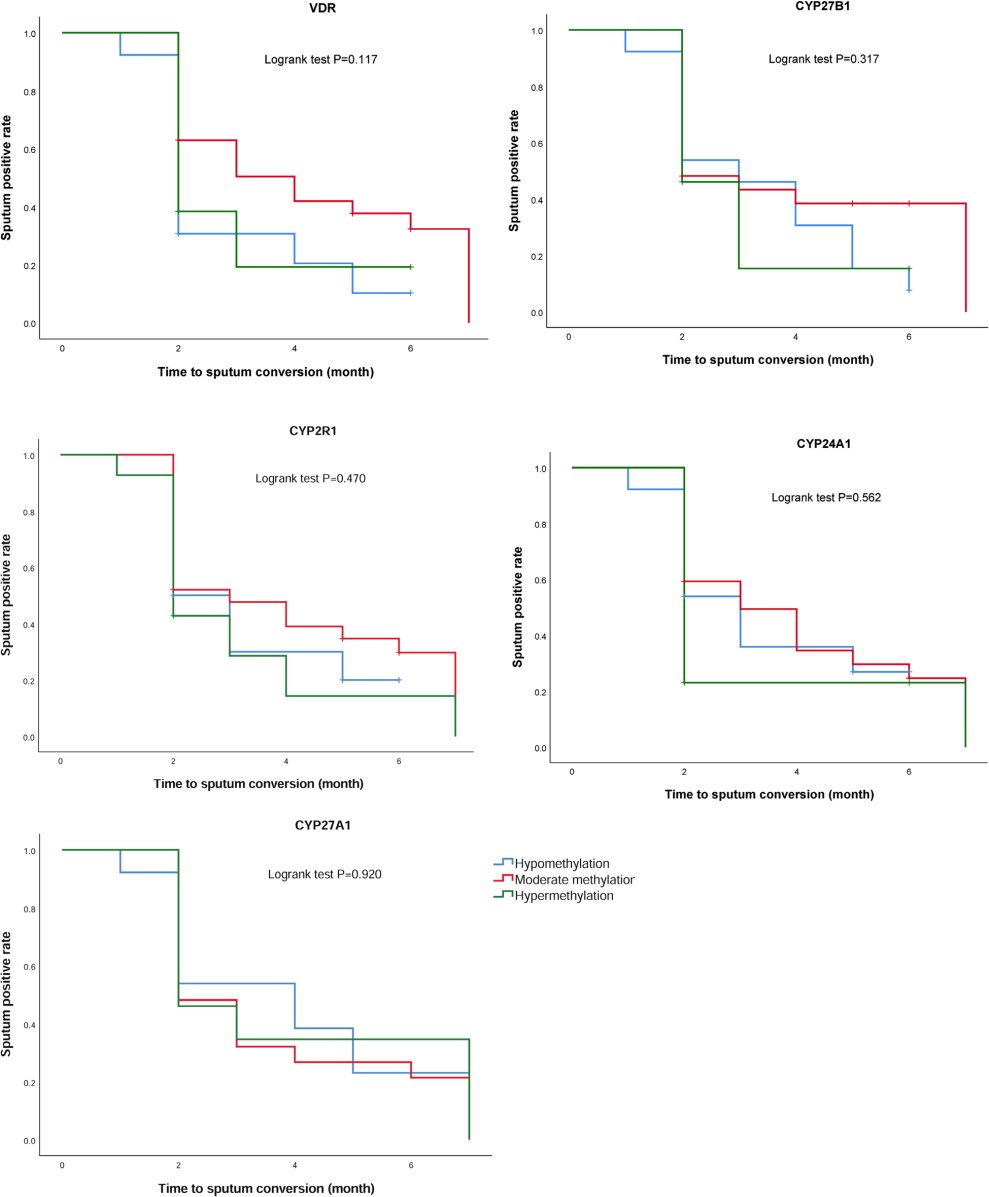
Time to sputum conversion in TB patients and cumulative methylation levels of genes in the vitamin D metabolic pathway. The cumulative methylation levels are categorized into three groups based on quartile. Hypomethylation, P_0_–P_25_. Moderate methylation, P_25_–P_75_. Hypermethylation, P_75_–P_100_

There were 23 CpG sites that were significantly related to the treatment outcomes (*P* < 0.05). The percentage of differentially methylated sites was 14.6% in the CYP24A1 gene, 2.7% in the CYP27A1 gene, 5.7% in the CYP27B1 gene, 5.9% in the CYP2R1 gene, and 10.7% in the VDR gene. However, no CpG sites remained significant after the Bonferroni correction. The cumulative methylation levels were categorized into three groups based on quartile (hypomethylation P_0_–P_25_; moderate methylation P_25_–P_75_; hypermethylation P_75_–P_100._). No significant relation was found between cumulative methylation level and sputum bacterium conversion (Fig. 2). We conducted crossover analysis and found no evidence of an interaction between CYP27A1 methylation level and 1,25(OH)_2_D concentration in sputum bacterium conversion (Table 10).

**Table 10:** Crossover analysis of CYP27A1 methylation levels and 1,25-dihydroxyvitamin D levels in the sputum conversion of patients with TB

1,25(OH)_2_D^a^	Cumulative methylation level^b^	Sputum conversion		OR	95% CI
		Yes	No		
Low	High	9	3	0.27	0.02–3.09
High	High	10	4	0.23	0.02–2.39
Low	Low	11	4	0.25	0.02–2.61
High	Low	11	1	1	

^a^Cutoff point of 1,25(OH)_2_D 79 pmol/L

^b^Cutoff point of cumulative methylation level of CYP27A1 0.435

## Discussion

Vitamins are being revisited for their role in pathogenicity and for their antimycobacterial properties. Vitamin C and vitamin D have been shown to possess antimycobacterial properties [9]. Previous studies have reported an association between vitamin D and immunity against TB [22, 23]. High levels of vitamin D can decrease the reactivation of latent TB and reduce the severity of active TB [24–27]. Vitamin D deficiency is believed to be a risk factor for the acquisition of TB infection [28, 29]. However, vitamin D supplementation to improve outcomes in TB patients has resulted in contradictory results [30, 31] that may be partly attributable to individual variation in vitamin D metabolic capacity and immunity. Previous studies have suggested that activation or silencing of certain signaling pathways plays a role in TB development [32]. Genetic information is carried not only in DNA sequences but also in epigenetic variations [33, 34]. In this study, we used next-generation sequencing to quantify the methylation levels of five vitamin D metabolic pathway genes and observed a significant association with the risk of TB.

Vitamin D has two main active metabolites: 25(OH)D and 1,25(OH)_2_D [35]. It binds to the vitamin D receptor on the membrane or cell nucleus to begin its activity in the transcription process [36]. In the present study, we observed that TB patients had significantly lower serum 1,25(OH)_2_D concentrations than controls and that the concentrations decreased after the initiation of antituberculosis therapy. 1,25(OH)_2_D is the biologically active form of vitamin D [37] and is associated with treatment outcomes of TB patients. The higher the 1,25(OH)_2_D concentration, the greater the likelihood of successful treatment and the higher the sputum negative conversion rate. Our findings support the hypothesis that we can assist TB treatment by increasing sunlight radiation and vitamin D intake. In a randomized double-blind placebo-controlled trial among 120 Mongolian children, vitamin D supplementation seemed to prevent the conversion of the tuberculin skin test [38]. However, another report suggested that the time to sputum conversion was not reduced with vitamin D supplementation [17].

VDR is essential for adequate immune function [22]. One study examined the methylation statuses of 17 CpGs in VDR and determined their relation to TB [39]. Our findings showed that hypomethylation of the VDR promoter may be a potential biomarker of TB, but its impact on VDR expression remains to be studied. CYP2R1 is a hepatic microsomal enzyme responsible for the 25-hydroxylation of vitamin D that is highly conserved among species ranging from fish to humans [40, 41]. CYP24A1 encodes 24-hydroxylase, which initiates the metabolism of both 25(OH)D and 1,25(OH)_2_D [40]. Zhou et al. conducted an intervention trial with vitamin D supplementation in postmenopausal women and reported that the baseline DNA methylation levels of CYP2R1 and CYP24A1 were negatively correlated with the concentrations of active 25(OH)D metabolite after vitamin D supplementation and that subjects with high DNA methylation levels needed higher vitamin D supplementation to reach optimal serum levels [42]. The CYP27A1 gene codes for 27-hydroxylase [43]. The methylation of CYP27A1 has been reported to be associated with the balance of bile acid in nonalcoholic fatty liver disease and in drug metabolism [44]. CYP27B1 catalyzes the de novo production of 1,25(OH)_2_D from accumulated 25(OH)D; 1,25(OH)_2_D is delivered to the cells via the vitamin D-binding protein (DBP), which is encoded by GC. The liganded VDR-transcription factor complex binds to vitamin D response elements (VDREs) in cathelicidin antimicrobial peptide (CAMP), activating CAMP expression [45]. The methylation level of CYP27B1 is elevated in primary lymphoma and leukemia cells [46].

There are several limitations in this study. First, we only measured the vitamin D levels at the baseline and at the end of the intensive treatment stage of TB patients; long-term continuous monitoring for the whole course of the disease may provide more information. Second, the regulation of gene transcription is complex, including genetic variation, modulation of the interactions of control factors with the transcriptional machinery, and epigenetic modification. These regulatory pathways do not function independently. Previous studies have reported methylation variation in association with genetic variation across individuals [47]. Genetic and epigenetic mechanisms may interact and together affect biological processes and disease development [48]. In this study, we only measured the methylation of the promoter regions of key genes in the vitamin D metabolic pathway. We noticed that the difference in methylation was subtle between cases and controls, although the *P* value was significant. Whether this subtle methylation difference could alter vitamin D metabolism and change macrophage M.tb killing capacity is not clear and needs more exploration. Other factors, such as genetic polymorphisms, sunlight exposure, food intake, and drug supplementation, can also affect vitamin D levels and the risk of TB [45]. Third, immune responses downstream of the vitamin D metabolic pathway should also be considered. Vitamin D has no direct antimycobacterial action, but its active metabolite 1,25(OH)_2_D modulates host responses to M.tb infection [49]. 1,25(OH)_2_D has been shown to induce antimycobacterial activity in macrophages in vitro, upregulate protective innate host responses, and trigger antimicrobial peptides such as cathelicidin [50]. M.tb entry into the body is mediated by macrophage toll-like receptors, which induce antibacterial autophagy by upregulating and activating VDR and increasing 1a-hydroxylase. Calcitriol activation of VDR induces CAMP gene expression and the subsequent production of cathelicidin, which disrupts the bacterial cell membrane and induces autophagy in monocytes [51]. Exogenous 1,25(OH)_2_D induces a superoxide burst and enhances phagolysosome fusion in M.tb-infected macrophages [49]. Simultaneously, the T cells secrete IFN-g, which promotes antimicrobial peptide expression, autophagy, phagosomal maturation, and antimicrobial action against M.tb within macrophages [51]. In this study, we did not evaluate the immune responses downstream of the vitamin D metabolic pathway. These immune responses should be considered in future studies.

In conclusion, our results suggest that the methylation levels of the CYP24A1, CYP27A1, CYP27B1, CYP2R1, and VDR genes in the metabolic pathway of vitamin D are related to the risk and prognosis of TB. Investigating the role of abnormal metabolism of vitamin D is important for the prevention and control of TB. Individualized vitamin D intervention based on epigenetic traits of key genes in its metabolic pathway will be valuable for the prevention and control of tuberculosis.

## Additional file

Additional file 1: Spearman’s correlation for methylation levels in each region with 1,25-dihydroxyvitamin D levels. (DOC 37 kb)

## Abbreviations

1,25(OH)_2_D: 1,25-dihydroxyvitamin D
25(OH)D: 25-hydroxyvitamin D
AUC: Area under the curve
BCG: Bacillus Calmette-Guérin
CAMP: Cathelicidin antimicrobial peptide
CI: Confidence interval
CV: Coefficient of variation
DBP: Vitamin D-binding protein
M.tb: *Mycobacterium tuberculosis*
OR: Odds ratio
ROC: Receiver operating characteristic
RR: Relative risk
SD: Standard deviation
TB: Tuberculosis
VDREs: Vitamin D response elements
