# Supplementary material for: Vitamin D and the promoter methylation of its metabolic pathway genes in association with the risk and prognosis of tuberculosis
Source: Clin Epigenetics. 2018 Sep 12;10:118. doi: 10.1186/s13148-018-0552-6 (PMC6136159; doi:10.1186/s13148-018-0552-6)
Supplement: Supplementary file 1 — Spearman’s correlation for methylation levels in each region with 1,25-dihydroxyvitamin D levels. (DOC 37 kb) [file 13148_2018_552_MOESM1_ESM.doc]

| Genes | Fragment | r | P |
| --- | --- | --- | --- |
| CYP24A1 | CYP24A1_1 | -0.032 | 0.619 |
|  | CYP24A1_2 | 0.069 | 0.284 |
| CYP27A1 | CYP27A1_1 | 0.060 | 0.351 |
|  | CYP27A1_2 | 0.082 | 0.206 |
|  | CYP27A1_3 | 0.129 | 0.045 |
|  | CYP27A1_4 | 0.065 | 0.319 |
|  | CYP27A1_5 | -0.021 | 0.749 |
| CYP27B1 | CYP27B1_1 | 0.016 | 0.805 |
|  | CYP27B1_2 | 0.121 | 0.060 |
|  | CYP27B1_3 | 0.032 | 0.618 |
| CYP2R1 | CYP2R1_1 | -0.035 | 0.591 |
|  | CYP2R1_2 | -0.052 | 0.427 |
|  | CYP2R1_3 | -0.045 | 0.489 |
|  | CYP2R1_4 | 0.069 | 0.286 |
| VDR | VDR _1 | 0.010 | 0.881 |
|  | VDR _2 | 0.104 | 0.109 |
|  | VDR _3 | 0.094 | 0.146 |
|  | VDR _4 | 0.022 | 0.739 |
|  | VDR _6 | -0.016 | 0.800 |

Additional file 1. Spearman's correlation for methylation levels in each region with 1,25-dihydroxyvitamin D levels
